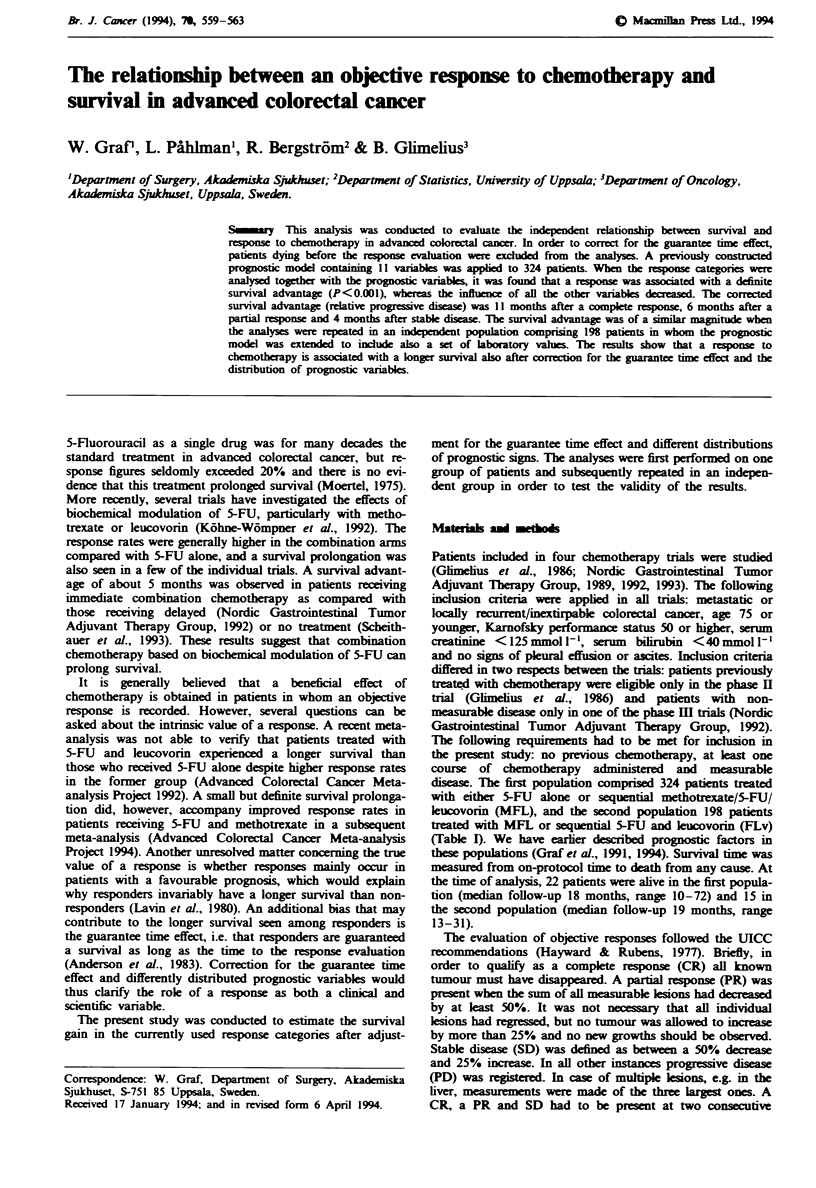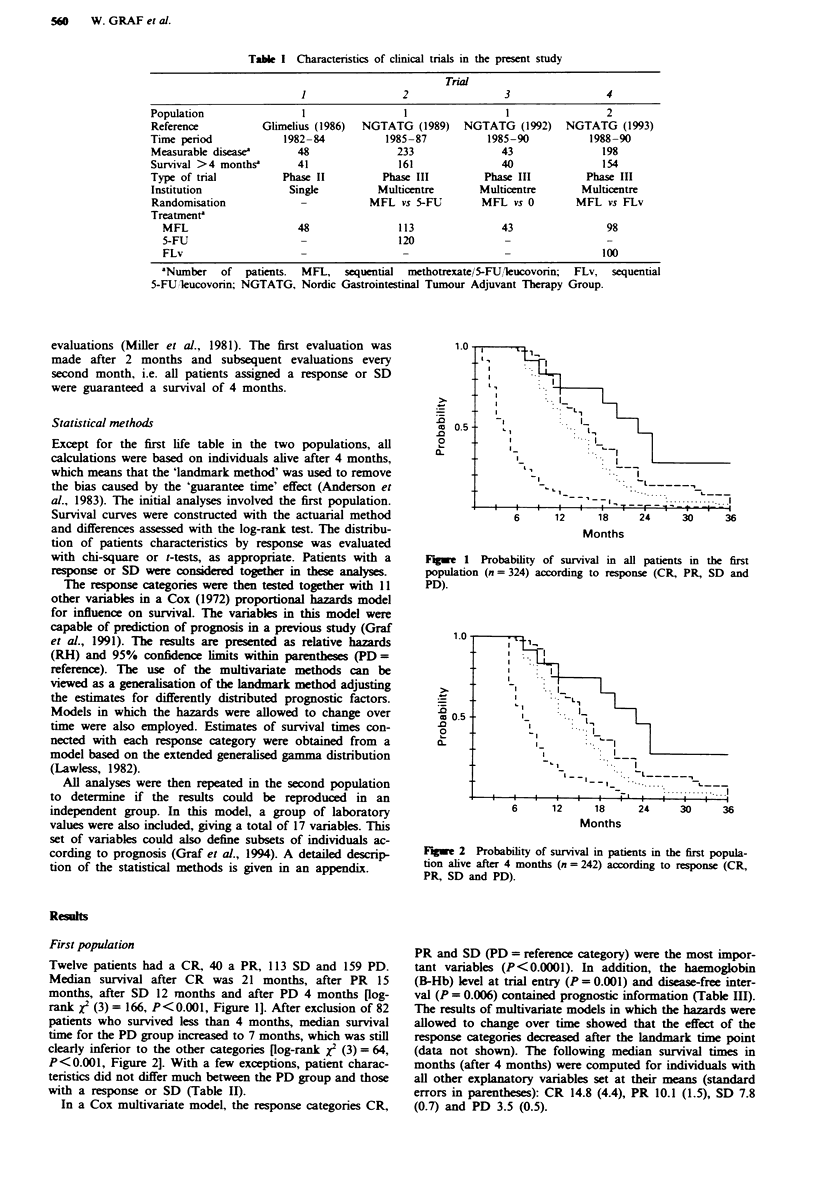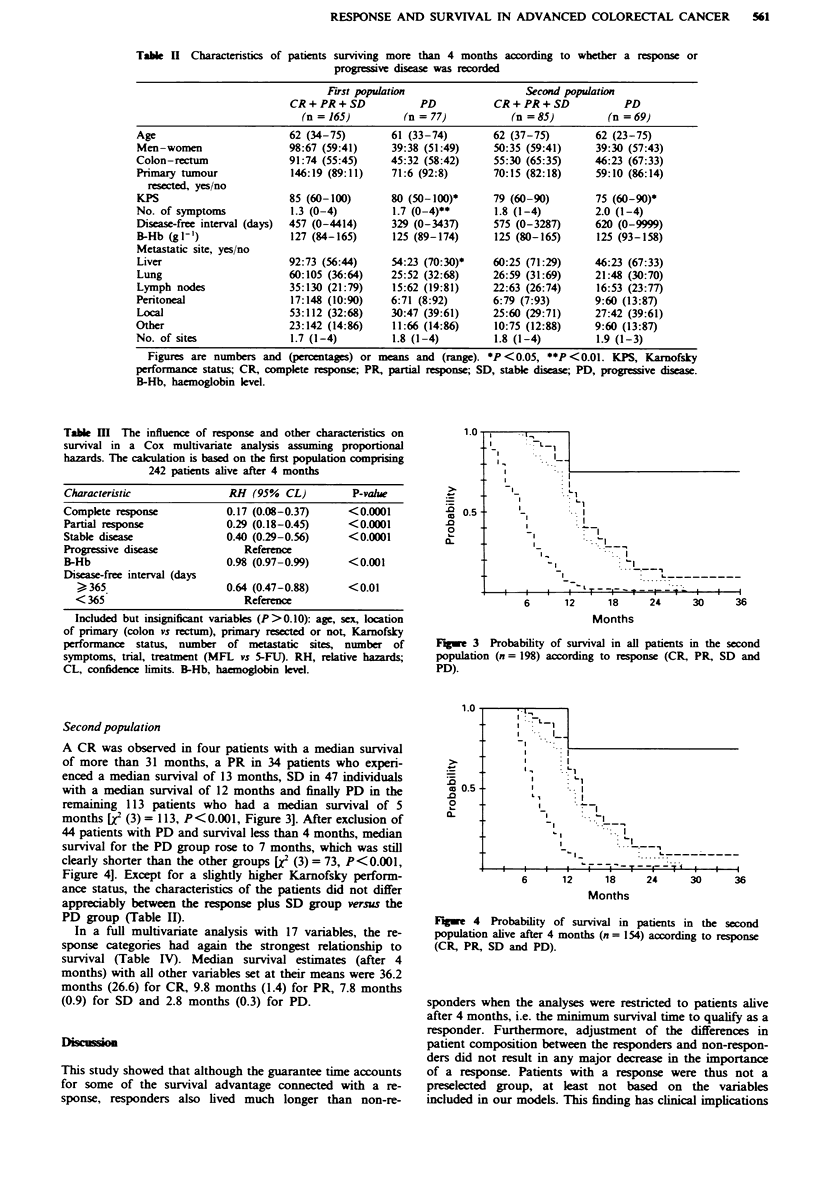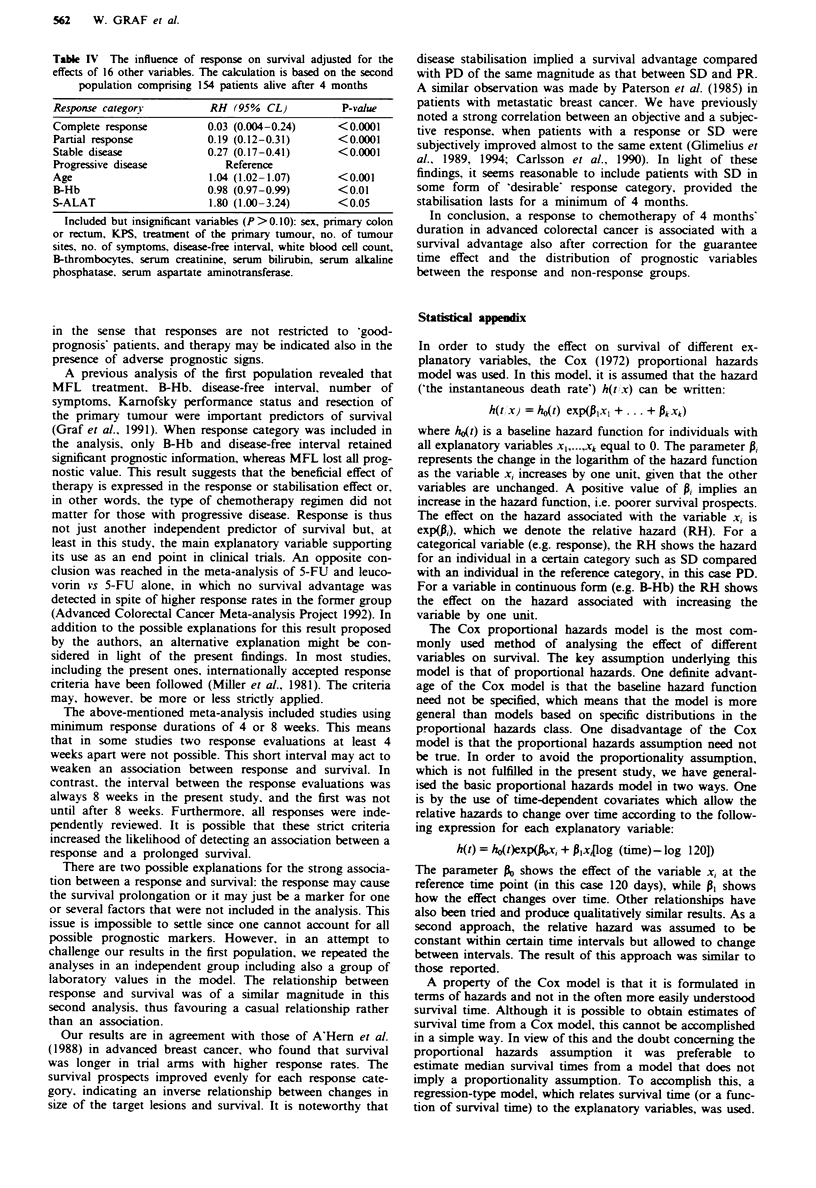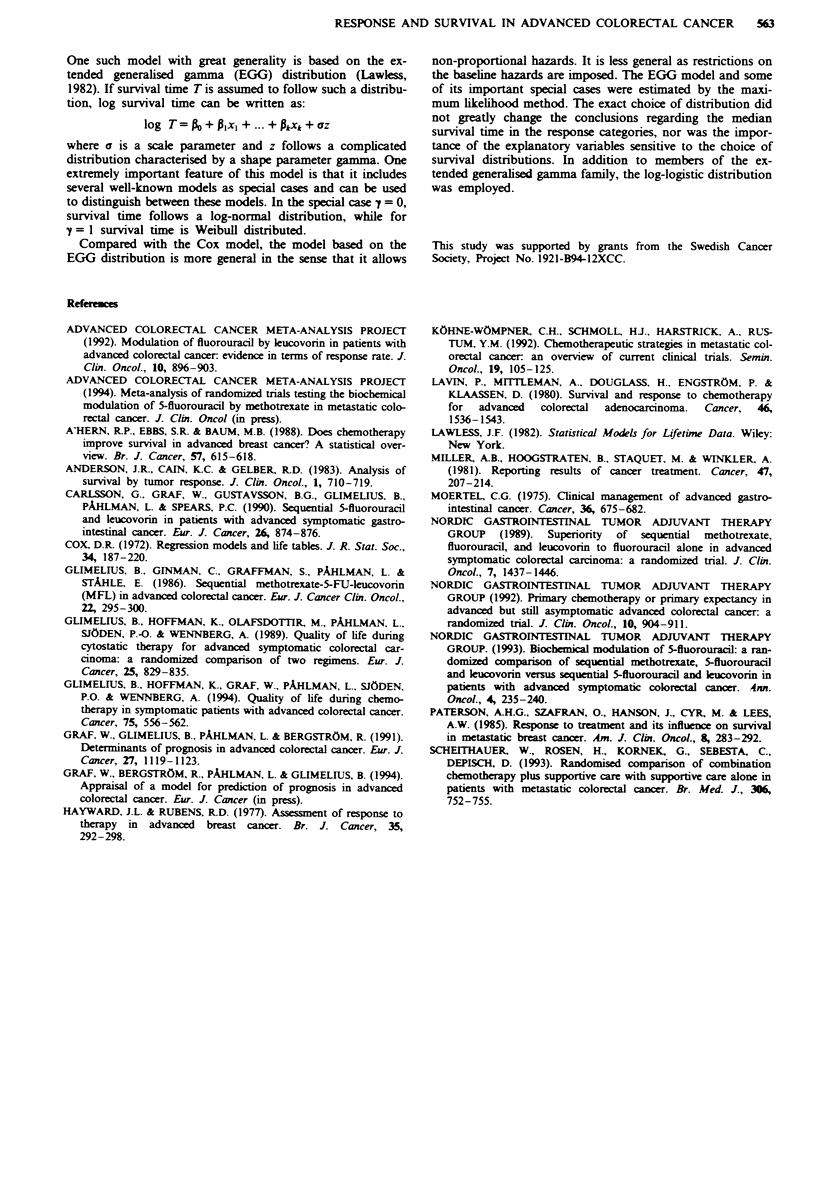# The relationship between an objective response to chemotherapy and survival in advanced colorectal cancer.

**DOI:** 10.1038/bjc.1994.345

**Published:** 1994-09

**Authors:** W. Graf, L. Påhlman, R. Bergström, B. Glimelius

**Affiliations:** Department of Surgery, Akademiska Sjukhuset, Uppsala, Sweden.

## Abstract

This analysis was conducted to evaluate the independent relationship between survival and response to chemotherapy in advanced colorectal cancer. In order to correct for the guarantee time effect, patients dying before the response evaluation were excluded from the analyses. A previously constructed prognostic model containing 11 variables was applied to 324 patients. When the response categories were analysed together with the prognostic variables, it was found that a response was associated with a definite survival advantage (P < 0.001), whereas the influence of all the other variables decreased. The corrected survival advantage (relative progressive disease) was 11 months after a complete response, 6 months after a partial response and 4 months after stable disease. The survival advantage was of a similar magnitude when the analyses were repeated in an independent population comprising 198 patients in whom the prognostic model was extended to include also a set of laboratory values. The results show that a response to chemotherapy is associated with a longer survival also after correction for the guarantee time effect and the distribution of prognostic variables.


Br. J. Cancer (1994), 70, 559-563 © Macmillan Press Ltd., 1994

### The relationship between an objective response to chemotherapy and survival in advanced colorectal cancer

W. Graf[1], L. Påhlman[1], R. Bergström[2] & B. Glimelius[3]

[1]*Department of Surgery, Akademiska Sjukhuset;* [2]*Department of Statistics, University of Uppsala;* [3]*Department of Oncology, Akademiska Sjukhuset, Uppsala, Sweden.*

**Summary** This analysis was conducted to evaluate the independent relationship between survival and response to chemotherapy in advanced colorectal cancer. In order to correct for the guarantee time effect, patients dying before the response evaluation were excluded from the analyses. A previously constructed prognostic model containing 11 variables was applied to 324 patients. When the response categories were analysed together with the prognostic variables, it was found that a response was associated with a definite survival advantage ($P<0.001$), whereas the influence of all the other variables decreased. The corrected survival advantage (relative progressive disease) was 11 months after a complete response, 6 months after a partial response and 4 months after stable disease. The survival advantage was of a similar magnitude when the analyses were repeated in an independent population comprising 198 patients in whom the prognostic model was extended to include also a set of laboratory values. The results show that a response to chemotherapy is associated with a longer survival also after correction for the guarantee time effect and the distribution of prognostic variables.

5-Fluorouracil as a single drug was for many decades the standard treatment in advanced colorectal cancer, but response figures seldomly exceeded 20% and there is no evidence that this treatment prolonged survival (Moertel, 1975). More recently, several trials have investigated the effects of biochemical modulation of 5-FU, particularly with methotrexate or leucovorin (Köhne-Wömpner *et al.*, 1992). The response rates were generally higher in the combination arms compared with 5-FU alone, and a survival prolongation was also seen in a few of the individual trials. A survival advantage of about 5 months was observed in patients receiving immediate combination chemotherapy as compared with those receiving delayed (Nordic Gastrointestinal Tumor Adjuvant Therapy Group, 1992) or no treatment (Scheithauer *et al.*, 1993). These results suggest that combination chemotherapy based on biochemical modulation of 5-FU can prolong survival.

It is generally believed that a beneficial effect of chemotherapy is obtained in patients in whom an objective response is recorded. However, several questions can be asked about the intrinsic value of a response. A recent meta-analysis was not able to verify that patients treated with 5-FU and leucovorin experienced a longer survival than those who received 5-FU alone despite higher response rates in the former group (Advanced Colorectal Cancer Meta-analysis Project 1992). A small but definite survival prolongation did, however, accompany improved response rates in patients receiving 5-FU and methotrexate in a subsequent meta-analysis (Advanced Colorectal Cancer Meta-analysis Project 1994). Another unresolved matter concerning the true value of a response is whether responses mainly occur in patients with a favourable prognosis, which would explain why responders invariably have a longer survival than non-responders (Lavin *et al.*, 1980). An additional bias that may contribute to the longer survival seen among responders is the guarantee time effect, i.e. that responders are guaranteed a survival as long as the time to the response evaluation (Anderson *et al.*, 1983). Correction for the guarantee time effect and differently distributed prognostic variables would thus clarify the role of a response as both a clinical and scientific variable.

The present study was conducted to estimate the survival gain in the currently used response categories after adjustment for the guarantee time effect and different distributions of prognostic signs. The analyses were first performed on one group of patients and subsequently repeated in an independent group in order to test the validity of the results.

Correspondence: W. Graf, Department of Surgery, Akademiska Sjukhuset, S-751 85 Uppsala, Sweden.
Received 17 January 1994; and in revised form 6 April 1994.

### Materials and methods

Patients included in four chemotherapy trials were studied (Glimelius *et al.*, 1986; Nordic Gastrointestinal Tumor Adjuvant Therapy Group, 1989, 1992, 1993). The following inclusion criteria were applied in all trials: metastatic or locally recurrent/inextirpable colorectal cancer, age 75 or younger, Karnofsky performance status 50 or higher, serum creatinine $<125\ \text{mmol}\ l^{-1}$, serum bilirubin $<40\ \text{mmol}\ l^{-1}$ and no signs of pleural effusion or ascites. Inclusion criteria differed in two respects between the trials: patients previously treated with chemotherapy were eligible only in the phase II trial (Glimelius *et al.*, 1986) and patients with non-measurable disease only in one of the phase III trials (Nordic Gastrointestinal Tumor Adjuvant Therapy Group, 1992). The following requirements had to be met for inclusion in the present study: no previous chemotherapy, at least one course of chemotherapy administered and measurable disease. The first population comprised 324 patients treated with either 5-FU alone or sequential methotrexate/5-FU/leucovorin (MFL), and the second population 198 patients treated with MFL or sequential 5-FU and leucovorin (FLv) (Table I). We have earlier described prognostic factors in these populations (Graf *et al.*, 1991, 1994). Survival time was measured from on-protocol time to death from any cause. At the time of analysis, 22 patients were alive in the first population (median follow-up 18 months, range 10-72) and 15 in the second population (median follow-up 19 months, range 13-31).

The evaluation of objective responses followed the UICC recommendations (Hayward & Rubens, 1977). Briefly, in order to qualify as a complete response (CR) all known tumour must have disappeared. A partial response (PR) was present when the sum of all measurable lesions had decreased by at least 50%. It was not necessary that all individual lesions had regressed, but no tumour was allowed to increase by more than 25% and no new growths should be observed. Stable disease (SD) was defined as between a 50% decrease and 25% increase. In all other instances progressive disease (PD) was registered. In case of multiple lesions, e.g. in the liver, measurements were made of the three largest ones. A CR, a PR and SD had to be present at two consecutive

**Table I** Characteristics of clinical trials in the present study

| | *Trial* 1 | 2 | 3 | 4 |
|---|---|---|---|---|
| Population | 1 | 1 | 1 | 2 |
| Reference | Glimelius (1986) | NGTATG (1989) | NGTATG (1992) | NGTATG (1993) |
| Time period | 1982-84 | 1985-87 | 1985-90 | 1988-90 |
| Measurable disease[a] | 48 | 233 | 43 | 198 |
| Survival >4 months[a] | 41 | 161 | 40 | 154 |
| Type of trial | Phase II | Phase III | Phase III | Phase III |
| Institution | Single | Multicentre | Multicentre | Multicentre |
| Randomisation | – | MFL *vs* 5-FU | MFL *vs* 0 | MFL *vs* FLv |
| Treatment[a] | | | | |
| MFL | 48 | 113 | 43 | 98 |
| 5-FU | – | 120 | – | – |
| FLv | – | – | – | 100 |

[a]Number of patients. MFL, sequential methotrexate/5-FU/leucovorin; FLv, sequential 5-FU/leucovorin; NGTATG, Nordic Gastrointestinal Tumour Adjuvant Therapy Group.

evaluations (Miller *et al.*, 1981). The first evaluation was made after 2 months and subsequent evaluations every second month, i.e. all patients assigned a response or SD were guaranteed a survival of 4 months.

### *Statistical methods*

Except for the first life table in the two populations, all calculations were based on individuals alive after 4 months, which means that the 'landmark method' was used to remove the bias caused by the 'guarantee time' effect (Anderson *et al.*, 1983). The initial analyses involved the first population. Survival curves were constructed with the actuarial method and differences assessed with the log-rank test. The distribution of patients characteristics by response was evaluated with chi-square or *t*-tests, as appropriate. Patients with a response or SD were considered together in these analyses.

The response categories were then tested together with 11 other variables in a Cox (1972) proportional hazards model for influence on survival. The variables in this model were capable of prediction of prognosis in a previous study (Graf *et al.*, 1991). The results are presented as relative hazards (RH) and 95% confidence limits within parentheses (PD = reference). The use of the multivariate methods can be viewed as a generalisation of the landmark method adjusting the estimates for differently distributed prognostic factors. Models in which the hazards were allowed to change over time were also employed. Estimates of survival times connected with each response category were obtained from a model based on the extended generalised gamma distribution (Lawless, 1982).

All analyses were then repeated in the second population to determine if the results could be reproduced in an independent group. In this model, a group of laboratory values were also included, giving a total of 17 variables. This set of variables could also define subsets of individuals according to prognosis (Graf *et al.*, 1994). A detailed description of the statistical methods is given in an appendix.

**Figure 1** Probability of survival in all patients in the first population ($n = 324$) according to response (CR, PR, SD and PD).

**Figure 2** Probability of survival in patients in the first population alive after 4 months ($n = 242$) according to response (CR, PR, SD and PD).

### Results

### *First population*

Twelve patients had a CR, 40 a PR, 113 SD and 159 PD. Median survival after CR was 21 months, after PR 15 months, after SD 12 months and after PD 4 months [log-rank $\chi^2$ (3) = 166, $P<0.001$, Figure 1]. After exclusion of 82 patients who survived less than 4 months, median survival time for the PD group increased to 7 months, which was still clearly inferior to the other categories [log-rank $\chi^2$ (3) = 64, $P<0.001$, Figure 2]. With a few exceptions, patient characteristics did not differ much between the PD group and those with a response or SD (Table II).

In a Cox multivariate model, the response categories CR, PR and SD (PD = reference category) were the most important variables ($P<0.0001$). In addition, the haemoglobin (B-Hb) level at trial entry ($P = 0.001$) and disease-free interval ($P = 0.006$) contained prognostic information (Table III). The results of multivariate models in which the hazards were allowed to change over time showed that the effect of the response categories decreased after the landmark time point (data not shown). The following median survival times in months (after 4 months) were computed for individuals with all other explanatory variables set at their means (standard errors in parentheses): CR 14.8 (4.4), PR 10.1 (1.5), SD 7.8 (0.7) and PD 3.5 (0.5).

**Table II** Characteristics of patients surviving more than 4 months according to whether a response or progressive disease was recorded

| | *First population* | | *Second population* | |
|---|---|---|---|---|
| | *CR + PR + SD* (n = *165*) | *PD* (n = *77*) | *CR + PR + SD* (n = *85*) | *PD* (n = *69*) |
| Age | 62 (34–75) | 61 (33–74) | 62 (37–75) | 62 (23–75) |
| Men–women | 98:67 (59:41) | 39:38 (51:49) | 50:35 (59:41) | 39:30 (57:43) |
| Colon–rectum | 91:74 (55:45) | 45:32 (58:42) | 55:30 (65:35) | 46:23 (67:33) |
| Primary tumour resected, yes/no | 146:19 (89:11) | 71:6 (92:8) | 70:15 (82:18) | 59:10 (86:14) |
| KPS | 85 (60–100) | 80 (50–100)* | 79 (60–90) | 75 (60–90)* |
| No. of symptoms | 1.3 (0–4) | 1.7 (0–4)** | 1.8 (1–4) | 2.0 (1–4) |
| Disease-free interval (days) | 457 (0–4414) | 329 (0–3437) | 575 (0–3287) | 620 (0–9999) |
| B-Hb (g $l^{-1}$) | 127 (84–165) | 125 (89–174) | 125 (80–165) | 125 (93–158) |
| Metastatic site, yes/no | | | | |
| Liver | 92:73 (56:44) | 54:23 (70:30)* | 60:25 (71:29) | 46:23 (67:33) |
| Lung | 60:105 (36:64) | 25:52 (32:68) | 26:59 (31:69) | 21:48 (30:70) |
| Lymph nodes | 35:130 (21:79) | 15:62 (19:81) | 22:63 (26:74) | 16:53 (23:77) |
| Peritoneal | 17:148 (10:90) | 6:71 (8:92) | 6:79 (7:93) | 9:60 (13:87) |
| Local | 53:112 (32:68) | 30:47 (39:61) | 25:60 (29:71) | 27:42 (39:61) |
| Other | 23:142 (14:86) | 11:66 (14:86) | 10:75 (12:88) | 9:60 (13:87) |
| No. of sites | 1.7 (1–4) | 1.8 (1–4) | 1.8 (1–4) | 1.9 (1–3) |

Figures are numbers and (percentages) or means and (range). *$P < 0.05$, **$P < 0.01$. KPS, Karnofsky performance status; CR, complete response; PR, partial response; SD, stable disease; PD, progressive disease. B-Hb, haemoglobin level.

**Table III** The influence of response and other characteristics on survival in a Cox multivariate analysis assuming proportional hazards. The calculation is based on the first population comprising 242 patients alive after 4 months

| *Characteristic* | *RH (95% CL)* | P-*value* |
|---|---|---|
| Complete response | 0.17 (0.08–0.37) | <0.0001 |
| Partial response | 0.29 (0.18–0.45) | <0.0001 |
| Stable disease | 0.40 (0.29–0.56) | <0.0001 |
| Progressive disease | Reference | |
| B-Hb | 0.98 (0.97–0.99) | <0.001 |
| Disease-free interval (days | | |
| ≥365 | 0.64 (0.47–0.88) | <0.01 |
| <365 | Reference | |

Included but insignificant variables ($P > 0.10$): age, sex, location of primary (colon *vs* rectum), primary resected or not, Karnofsky performance status, number of metastatic sites, number of symptoms, trial, treatment (MFL *vs* 5-FU). RH, relative hazards; CL, confidence limits. B-Hb, haemoglobin level.

**Figure 3** Probability of survival in all patients in the second population ($n = 198$) according to response (CR, PR, SD and PD).

**Figure 4** Probability of survival in patients in the second population alive after 4 months ($n = 154$) according to response (CR, PR, SD and PD).

*Second population*

A CR was observed in four patients with a median survival of more than 31 months, a PR in 34 patients who experienced a median survival of 13 months, SD in 47 individuals with a median survival of 12 months and finally PD in the remaining 113 patients who had a median survival of 5 months [$\chi^2$ (3) = 113, $P < 0.001$, Figure 3]. After exclusion of 44 patients with PD and survival less than 4 months, median survival for the PD group rose to 7 months, which was still clearly shorter than the other groups [$\chi^2$ (3) = 73, $P < 0.001$, Figure 4]. Except for a slightly higher Karnofsky performance status, the characteristics of the patients did not differ appreciably between the response plus SD group *versus* the PD group (Table II).

In a full multivariate analysis with 17 variables, the response categories had again the strongest relationship to survival (Table IV). Median survival estimates (after 4 months) with all other variables set at their means were 36.2 months (26.6) for CR, 9.8 months (1.4) for PR, 7.8 months (0.9) for SD and 2.8 months (0.3) for PD.

### Discussion

This study showed that although the guarantee time accounts for some of the survival advantage connected with a response, responders also lived much longer than non-responders when the analyses were restricted to patients alive after 4 months, i.e. the minimum survival time to qualify as a responder. Furthermore, adjustment of the differences in patient composition between the responders and non-responders did not result in any major decrease in the importance of a response. Patients with a response were thus not a preselected group, at least not based on the variables included in our models. This finding has clinical implications

Table IV The influence of response on survival adjusted for the effects of 16 other variables. The calculation is based on the second population comprising 154 patients alive after 4 months

| Response category | RH (95% CL) | P-value |
|---|---|---|
| Complete response | 0.03 (0.004–0.24) | <0.0001 |
| Partial response | 0.19 (0.12–0.31) | <0.0001 |
| Stable disease | 0.27 (0.17–0.41) | <0.0001 |
| Progressive disease | Reference | |
| Age | 1.04 (1.02–1.07) | <0.001 |
| B-Hb | 0.98 (0.97–0.99) | <0.01 |
| S-ALAT | 1.80 (1.00–3.24) | <0.05 |

Included but insignificant variables ($P > 0.10$): sex, primary colon or rectum, KPS, treatment of the primary tumour, no. of tumour sites, no. of symptoms, disease-free interval, white blood cell count, B-thrombocytes, serum creatinine, serum bilirubin, serum alkaline phosphatase, serum aspartate aminotransferase.

in the sense that responses are not restricted to 'good-prognosis' patients, and therapy may be indicated also in the presence of adverse prognostic signs.

A previous analysis of the first population revealed that MFL treatment, B-Hb, disease-free interval, number of symptoms, Karnofsky performance status and resection of the primary tumour were important predictors of survival (Graf et al., 1991). When response category was included in the analysis, only B-Hb and disease-free interval retained significant prognostic information, whereas MFL lost all prognostic value. This result suggests that the beneficial effect of therapy is expressed in the response or stabilisation effect or, in other words, the type of chemotherapy regimen did not matter for those with progressive disease. Response is thus not just another independent predictor of survival but, at least in this study, the main explanatory variable supporting its use as an end point in clinical trials. An opposite conclusion was reached in the meta-analysis of 5-FU and leucovorin vs 5-FU alone, in which no survival advantage was detected in spite of higher response rates in the former group (Advanced Colorectal Cancer Meta-analysis Project 1992). In addition to the possible explanations for this result proposed by the authors, an alternative explanation might be considered in light of the present findings. In most studies, including the present ones, internationally accepted response criteria have been followed (Miller et al., 1981). The criteria may, however, be more or less strictly applied.

The above-mentioned meta-analysis included studies using minimum response durations of 4 or 8 weeks. This means that in some studies two response evaluations at least 4 weeks apart were not possible. This short interval may act to weaken an association between response and survival. In contrast, the interval between the response evaluations was always 8 weeks in the present study, and the first was not until after 8 weeks. Furthermore, all responses were independently reviewed. It is possible that these strict criteria increased the likelihood of detecting an association between a response and a prolonged survival.

There are two possible explanations for the strong association between a response and survival: the response may cause the survival prolongation or it may just be a marker for one or several factors that were not included in the analysis. This issue is impossible to settle since one cannot account for all possible prognostic markers. However, in an attempt to challenge our results in the first population, we repeated the analyses in an independent group including also a group of laboratory values in the model. The relationship between response and survival was of a similar magnitude in this second analysis, thus favouring a casual relationship rather than an association.

Our results are in agreement with those of A'Hern et al. (1988) in advanced breast cancer, who found that survival was longer in trial arms with higher response rates. The survival prospects improved evenly for each response category, indicating an inverse relationship between changes in size of the target lesions and survival. It is noteworthy that disease stabilisation implied a survival advantage compared with PD of the same magnitude as that between SD and PR. A similar observation was made by Paterson et al. (1985) in patients with metastatic breast cancer. We have previously noted a strong correlation between an objective and a subjective response, when patients with a response or SD were subjectively improved almost to the same extent (Glimelius et al., 1989, 1994; Carlsson et al., 1990). In light of these findings, it seems reasonable to include patients with SD in some form of 'desirable' response category, provided the stabilisation lasts for a minimum of 4 months.

In conclusion, a response to chemotherapy of 4 months' duration in advanced colorectal cancer is associated with a survival advantage also after correction for the guarantee time effect and the distribution of prognostic variables between the response and non-response groups.

### Statistical appendix

In order to study the effect on survival of different explanatory variables, the Cox (1972) proportional hazards model was used. In this model, it is assumed that the hazard ('the instantaneous death rate') $h(t/x)$ can be written:

$$h(t/x) = h_0(t)\ \exp(\beta_1 x_1 + \ldots + \beta_k x_k)$$

where $h_0(t)$ is a baseline hazard function for individuals with all explanatory variables $x_1,\ldots,x_k$ equal to 0. The parameter $\beta_i$ represents the change in the logarithm of the hazard function as the variable $x_i$ increases by one unit, given that the other variables are unchanged. A positive value of $\beta_i$ implies an increase in the hazard function, i.e. poorer survival prospects. The effect on the hazard associated with the variable $x_i$ is $\exp(\beta_i)$, which we denote the relative hazard (RH). For a categorical variable (e.g. response), the RH shows the hazard for an individual in a certain category such as SD compared with an individual in the reference category, in this case PD. For a variable in continuous form (e.g. B-Hb) the RH shows the effect on the hazard associated with increasing the variable by one unit.

The Cox proportional hazards model is the most commonly used method of analysing the effect of different variables on survival. The key assumption underlying this model is that of proportional hazards. One definite advantage of the Cox model is that the baseline hazard function need not be specified, which means that the model is more general than models based on specific distributions in the proportional hazards class. One disadvantage of the Cox model is that the proportional hazards assumption need not be true. In order to avoid the proportionality assumption, which is not fulfilled in the present study, we have generalised the basic proportional hazards model in two ways. One is by the use of time-dependent covariates which allow the relative hazards to change over time according to the following expression for each explanatory variable:

$$h(t) = h_0(t)\exp(\beta_0 x_i + \beta_1 x_i[\log\ (\text{time}) - \log\ 120])$$

The parameter $\beta_0$ shows the effect of the variable $x_i$ at the reference time point (in this case 120 days), while $\beta_1$ shows how the effect changes over time. Other relationships have also been tried and produce qualitatively similar results. As a second approach, the relative hazard was assumed to be constant within certain time intervals but allowed to change between intervals. The result of this approach was similar to those reported.

A property of the Cox model is that it is formulated in terms of hazards and not in the often more easily understood survival time. Although it is possible to obtain estimates of survival time from a Cox model, this cannot be accomplished in a simple way. In view of this and the doubt concerning the proportional hazards assumption it was preferable to estimate median survival times from a model that does not imply a proportionality assumption. To accomplish this, a regression-type model, which relates survival time (or a function of survival time) to the explanatory variables, was used.

One such model with great generality is based on the extended generalised gamma (EGG) distribution (Lawless, 1982). If survival time $T$ is assumed to follow such a distribution, log survival time can be written as:

$$\log T = \beta_0 + \beta_1 x_1 + \ldots + \beta_k x_k + \sigma z$$

where $\sigma$ is a scale parameter and $z$ follows a complicated distribution characterised by a shape parameter gamma. One extremely important feature of this model is that it includes several well-known models as special cases and can be used to distinguish between these models. In the special case $\gamma = 0$, survival time follows a log-normal distribution, while for $\gamma = 1$ survival time is Weibull distributed.

Compared with the Cox model, the model based on the EGG distribution is more general in the sense that it allows non-proportional hazards. It is less general as restrictions on the baseline hazards are imposed. The EGG model and some of its important special cases were estimated by the maximum likelihood method. The exact choice of distribution did not greatly change the conclusions regarding the median survival time in the response categories, nor was the importance of the explanatory variables sensitive to the choice of survival distributions. In addition to members of the extended generalised gamma family, the log-logistic distribution was employed.

This study was supported by grants from the Swedish Cancer Society, Project No. 1921-B94-12XCC.

**References**

ADVANCED COLORECTAL CANCER META-ANALYSIS PROJECT (1992). Modulation of fluorouracil by leucovorin in patients with advanced colorectal cancer: evidence in terms of response rate. *J. Clin. Oncol.*, **10**, 896–903.

ADVANCED COLORECTAL CANCER META-ANALYSIS PROJECT (1994). Meta-analysis of randomized trials testing the biochemical modulation of 5-fluorouracil by methotrexate in metastatic colorectal cancer. *J. Clin. Oncol* (in press).

A'HERN, R.P., EBBS, S.R. & BAUM, M.B. (1988). Does chemotherapy improve survival in advanced breast cancer? A statistical overview. *Br. J. Cancer*, **57**, 615–618.

ANDERSON, J.R., CAIN, K.C. & GELBER, R.D. (1983). Analysis of survival by tumor response. *J. Clin. Oncol.*, **1**, 710–719.

CARLSSON, G., GRAF, W., GUSTAVSSON, B.G., GLIMELIUS, B., PÅHLMAN, L. & SPEARS, P.C. (1990). Sequential 5-fluorouracil and leucovorin in patients with advanced symptomatic gastrointestinal cancer. *Eur. J. Cancer*, **26**, 874–876.

COX, D.R. (1972). Regression models and life tables. *J. R. Stat. Soc.*, **34**, 187–220.

GLIMELIUS, B., GINMAN, C., GRAFFMAN, S., PÅHLMAN, L. & STÅHLE, E. (1986). Sequential methotrexate-5-FU-leucovorin (MFL) in advanced colorectal cancer. *Eur. J. Cancer Clin. Oncol.*, **22**, 295–300.

GLIMELIUS, B., HOFFMAN, K., OLAFSDOTTIR, M., PÅHLMAN, L., SJÖDEN, P.-O. & WENNBERG, A. (1989). Quality of life during cytostatic therapy for advanced symptomatic colorectal carcinoma: a randomized comparison of two regimens. *Eur. J. Cancer*, **25**, 829–835.

GLIMELIUS, B., HOFFMAN, K., GRAF, W., PÅHLMAN, L., SJÖDEN, P.O. & WENNBERG, A. (1994). Quality of life during chemotherapy in symptomatic patients with advanced colorectal cancer. *Cancer*, **75**, 556–562.

GRAF, W., GLIMELIUS, B., PÅHLMAN, L. & BERGSTRÖM, R. (1991). Determinants of prognosis in advanced colorectal cancer. *Eur. J. Cancer*, **27**, 1119–1123.

GRAF, W., BERGSTRÖM, R., PÅHLMAN, L. & GLIMELIUS, B. (1994). Appraisal of a model for prediction of prognosis in advanced colorectal cancer. *Eur. J. Cancer* (in press).

HAYWARD, J.L. & RUBENS, R.D. (1977). Assessment of response to therapy in advanced breast cancer. *Br. J. Cancer*, **35**, 292–298.

KÖHNE-WÖMPNER, C.H., SCHMOLL, H.J., HARSTRICK, A., RUSTUM, Y.M. (1992). Chemotherapeutic strategies in metastatic colorectal cancer: an overview of current clinical trials. *Semin. Oncol.*, **19**, 105–125.

LAVIN, P., MITTLEMAN, A., DOUGLASS, H., ENGSTRÖM, P. & KLAASSEN, D. (1980). Survival and response to chemotherapy for advanced colorectal adenocarcinoma. *Cancer*, **46**, 1536–1543.

LAWLESS, J.F. (1982). *Statistical Models for Lifetime Data*. Wiley: New York.

MILLER, A.B., HOOGSTRATEN, B., STAQUET, M. & WINKLER, A. (1981). Reporting results of cancer treatment. *Cancer*, **47**, 207–214.

MOERTEL, C.G. (1975). Clinical management of advanced gastrointestinal cancer. *Cancer*, **36**, 675–682.

NORDIC GASTROINTESTINAL TUMOR ADJUVANT THERAPY GROUP (1989). Superiority of sequential methotrexate, fluorouracil, and leucovorin to fluorouracil alone in advanced symptomatic colorectal carcinoma: a randomized trial. *J. Clin. Oncol.*, **7**, 1437–1446.

NORDIC GASTROINTESTINAL TUMOR ADJUVANT THERAPY GROUP (1992). Primary chemotherapy or primary expectancy in advanced but still asymptomatic advanced colorectal cancer: a randomized trial. *J. Clin. Oncol.*, **10**, 904–911.

NORDIC GASTROINTESTINAL TUMOR ADJUVANT THERAPY GROUP. (1993). Biochemical modulation of 5-fluorouracil: a randomized comparison of sequential methotrexate, 5-fluorouracil and leucovorin versus sequential 5-fluorouracil and leucovorin in patients with advanced symptomatic colorectal cancer. *Ann. Oncol.*, **4**, 235–240.

PATERSON, A.H.G., SZAFRAN, O., HANSON, J., CYR, M. & LEES, A.W. (1985). Response to treatment and its influence on survival in metastatic breast cancer. *Am. J. Clin. Oncol.*, **8**, 283–292.

SCHEITHAUER, W., ROSEN, H., KORNEK, G., SEBESTA, C., DEPISCH, D. (1993). Randomised comparison of combination chemotherapy plus supportive care with supportive care alone in patients with metastatic colorectal cancer. *Br. Med. J.*, **306**, 752–755.